# Population genetics of plant fungal threats: Insights from wheat powdery mildew

**DOI:** 10.1371/journal.pbio.3003151

**Published:** 2025-05-05

**Authors:** Sergio M. Latorre

**Affiliations:** Centre for Life’s Origins and Evolution, Department of Genetics, Evolution and Environment, University College London, London, United Kingdom

## Abstract

Population genetics studies of microbial pathogens are demonstrating the potential of evolutionary theory to inform decisions that impact society. This Primer explores a recent study on wheat powdery mildew fungus in PLOS Biology which uses genomic data to reveal the pathogen’s population structure and to predict its continental-scale dispersion routes.

## Teaser

Population genetics studies of microbial pathogens are demonstrating the potential of evolutionary theory to inform decisions that impact society. A recent study on wheat powdery mildew fungus in PLOS Biology uses genomic data to reveal the pathogen’s population structure and to predict its continental-scale dispersion routes.

Population genetics studies of fungal, bacterial, and viral pathogens have effectively demonstrated the potential to bridge the gap between evolutionary theory and scientifically informed decision-making that impacts society. This field has accelerated the translation of fundamental evolutionary principles into actionable strategies in diverse areas, including hospital protocols, trade policies, vaccination programs, and agricultural practices. For instance, insights from population genetics have informed the development of targeted vaccines, optimized antibiotic stewardship, and the management of crop diseases [1]. At the same time, the modern surge in pandemics and the rapid expansion of microbial pathogens, coupled with increasingly accessible technological advancements, are generating high loads of empirical data. These data are challenging and refining our understanding of classical evolutionary theory, revealing nuances in pathogen adaptation, transmission dynamics, and host–pathogen interactions that were previously overlooked.

Fungal microbial pathogens are an exemplary case study, as they pose a profound and escalating threat to global food security. These pathogens can cause significant crop losses, reduce agricultural productivity, and destabilize food supply chains, particularly in regions reliant on staple crops. Because of their importance, plant pathologists have relied on morphological, physiological, and virulence markers as swift and effective methods to identify and describe pathogenic clades and lineages of agricultural importance [2]. These traditional approaches have undoubtedly contributed to the development of effective disease management strategies, such as the breeding of resistant crop varieties and the application of targeted fungicides.

However, while these methods have been useful, they have important limitations. Many of the markers used in traditional pathogen identification are under strong selection pressures, which can lead to biased interpretations of pathogen diversity and evolution. This is the case of virulence markers which are often subject to rapid evolutionary changes due to the host–pathogen *arms race* or morphological and physiological traits which can be influenced by environmental conditions, making them unreliable for inferring long-term evolutionary trends [3].

To address these limitations, it has now become obvious that incorporating whole-genome data into the study of fungal plant pathogens is essential, as it involves markers under different selective regimes, including neutral markers [4]. It has been shown how the use of such genomic information can lead to the identification of key virulence factors, such as effector proteins, that play critical roles in host–pathogen interactions, or facilitat the phylogenetic reconstruction of relationships among pathogen populations, shedding light on their evolutionary origins and histories as well as their diversification patterns. Moreover, different population genetics studies have revealed the importance of recombination, gene flow, mobile genetic elements, and hybridization in generating genetic diversity and facilitating the emergence of new pathogenic strains and lineages [5].

*Blumeria graminis forma specialis tritici* (Bgt), the causal agent of wheat powdery mildew, exemplifies many of the hallmarks that make fungal pathogens a menace to food security: (*i*) it infects wheat, one of the world’s most widely cultivated staple crops [6]; (ii) its relatively short generation time enables it to accumulate genetic variation rapidly, increasing its potential to evade plant resistance mechanisms and develop fungicide resistance; (iii) its capacity for sexual reproduction further enhances genetic diversity, facilitating adaptation to changing environmental conditions and control measures; (iv) and its ability to produce a large number of spores ensures high census populations within crops, amplifying its spread, persistence and adaptation potential in agricultural systems [7,8].

Now, in this issue of PLOS Biology, Jigisha and colleagues [9] recently published an innovative research article utilizing a comprehensive whole-genomic-scale dataset comprising 415 Bgt isolates. These isolates were collected from various countries across Europe and surrounding regions over a span of nearly four decades, providing a robust temporal and geographical representation of the pathogen’s population. What distinguishes their work is not only the scale and diversity of their dataset but also their creative yet rigorous application of population genetics methods and genomic analyses to address critical questions in plant pathology. By examining patterns of genomic diversity in the isolates, Jigisha and colleagues were able to infer several key aspects of Bgt’s epidemiology and evolutionary history. They mapped the extent and direction of geographical dispersal routes, revealing patterns of pathogen movement across regions. Additionally, their analysis highlighted the connectedness of epidemics in different areas, showing how regional outbreaks may influence one another. Finally, they estimated the shifts between sexual and asexual reproduction, providing insights into the evolutionary dynamics and adaptive potential of the pathogen.

Jigisha and colleagues found a highly connected and genetically homogeneous population dominating Northern Europe, in contrast to smaller, more localized populations in Southern Europe that exhibited less interconnectedness. This regional differentiation highlights the influence of environmental and ecological factors on pathogen population structure. The authors elegantly demonstrated how this population structure correlates with prevailing wind patterns, suggesting that meteorological data could be leveraged to predict demographic dynamics and migration routes of this important pathogen. While some pathogenic fungi rely on alternative dispersal mechanisms, such as vectors, rain splash, or trade-related movement of plant material, this finding opens new avenues for integrating climate and weather data into predictive models. Such integration could improve early warning systems and targeted interventions for wheat powdery mildew management, as has already been shown in the specific case of rust fungi [10].

Furthermore, the study identified specific haplotypes of the fungal effector *AvrPm17*, which have undergone a selective sweep on standing genetic variation. These haplotypes rapidly increased in frequency successfully evading plant resistance mechanisms across multiple regions of the continent. This alarming trend highlights the pathogen’s ability to adapt quickly to host defenses, a process likely driven by the large census population size, despite the relatively low overall genetic diversity in Btg populations. These findings raise urgent concerns for the development of new resistant wheat varieties.

In summary, this work demonstrates how harnessing fine-grained population-level knowledge can provide practical solutions for monitoring, preventing, and facing increasingly common agricultural fungal threats like Bgt.
